# Sensorimotor delays in tracking may be compensated by negative feedback control of motion-extrapolated position

**DOI:** 10.1007/s00221-020-05962-0

**Published:** 2020-11-02

**Authors:** Maximilian G. Parker, Andrew P. Weightman, Sarah F. Tyson, Bruce Abbott, Warren Mansell

**Affiliations:** 1 Department of Psychology, University of Cambridge, Downing Street, Cambridge, CB2 3EB UK; 2 Division of Mechanical, Aerospace and Civil Engineering, The University of Manchester, Manchester, UK; 3 Division of Nursing, Midwifery and Social Work, The University of Manchester, Manchester, UK; 4 Psychology Department, Purdue University, Fort Wayne, IN USA; 5 Division of Psychology and Mental Health, University of Manchester, Manchester, UK

**Keywords:** Sensorimotor delay, Pursuit tracking, Action control

## Abstract

Sensorimotor delays dictate that humans act on outdated perceptual information. As a result, continuous manual tracking of an unpredictable target incurs significant response delays. However, no such delays are observed for repeating targets such as the sinusoids. Findings of this kind have led researchers to claim that the nervous system constructs predictive, probabilistic models of the world. However, a more parsimonious explanation is that visual perception of a moving target position is systematically biased by its velocity. The resultant extrapolated position could be compared with the cursor position and the difference canceled by negative feedback control, compensating sensorimotor delays. The current study tested whether a position extrapolation model fit human tracking of sinusoid (predictable) and pseudorandom (less predictable) targets better than the non-biased position control model, Twenty-eight participants tracked these targets and the two computational models were fit to the data at 60 fixed loop delay values (simulating sensorimotor delays). We observed that pseudorandom targets were tracked with a significantly greater phase delay than sinusoid targets. For sinusoid targets, the position extrapolation model simulated tracking results more accurately for loop delays longer than 120 ms, thereby confirming its ability to compensate for sensorimotor delays. However, for pseudorandom targets, this advantage arose only after 300 ms, indicating that velocity information is unlikely to be exploited in this way during the tracking of less predictable targets. We conclude that negative feedback control of position is a parsimonious model for tracking pseudorandom targets and that negative feedback control of extrapolated position is a parsimonious model for tracking sinusoidal targets.

## Introduction

Sensorimotor delays accumulate as a result of delays in afferent and efferent signal transmission, and central processing (Carlton 1981; Smith and Bowen 1980; Smith et al. 1960). Consequently, humans act on outdated sensory inputs (Carlton 1981; Stepp 2009; Wolpert et al. 2001). This delay presents a significant issue in action control, for ‘if the system is changing rapidly, then by the time a feedback signal has been used to modify the motor commands, the system will have evolved to a new state for which the corrective signal is inappropriate’ (Hollerbach 1982). The central nervous system (CNS) must therefore anticipate and compensate for the deleterious effect of delays to movements in time-sensitive tasks such as object interception and avoidance and driving. Such compensation can be observed readily in the ‘flash-lag’ effect, an illusion in which a continuously moving target is observed to be advanced in position to a sudden onset target presented in-phase (Nijhawan 2002). The illusion may be explained by a tendency for humans to extrapolate target motion to mitigate sensorimotor delays in perception.

Feedback delays in movement vary in magnitude throughout the CNS, but generally increase as a function of the length of the feedback loop and the number of local networks employed. On the shortest timescales, tactile feedback in the form of the muscle stretch and spinal reflexes may operate within tens of milliseconds (Sloot et al. 2015). Feedback delays for visuo-manual responses are substantially longer. Retinal information is projected to the primary visual cortex from the lateral geniculate nucleus (LGN) within about 30 ms (Foxe and Simpson 2002) and peaks around 60 ms post stimulus onset (Kruse et al. 2002). Activation onsets in the middle temporal (MT) and medial superior temporal (MST) cells occur around 40 ms post stimulus onset. These areas encode stimulus direction and velocity, and are considered the gateway to the dorsal stream (‘where’ pathway). In the dorsal stream, the MT and MST areas project to frontal areas (executive control) and the posterior parietal cortex (PPC), which has been implicated in coding feedback-dependent computations of movement error, which are used to correct movement trajectories online (Desmurget et al. 1999; Gréa et al. 2002). Activation onset in the PPC occurs at approximately 80 ms and peaks after 100 ms. The PPC projects to the Supplementary Motor Area (SMA) and to the motor cortices. Recurrent activation occurs throughout the dorsal stream during movement preparation and execution, such that “100–400 ms is commonly needed for information processing prior to response output in humans.” (Foxe and Simpson 2002). Reaching experiments in which the location of the target is changed during an ongoing movement toward it corroborate electrophysiological estimates of feedback delays. Trajectory corrections can be made on the basis visual feedback of the new target location within 100–150 ms (Brenner and Smeets 2015; Day and Lyon 2000; Foulkes and Miall 2000; Franklin and Wolpert 2008; Saunders and Knill 2005). Thus, 100 ms represents a plausible minimum estimate for sensorimotor feedback delay in visuo-manual tasks.

During sustained tracking of a moving target, manual tracking, response delays can be measured continuously as the phase lag between the target and cursor. Interestingly, these phase lags vary widely depending on target characteristics. Under the right conditions, lags can be measured that are shorter than the minimal estimates of visuo-manual feedback delay and must therefore represent a mitigation of sensorimotor delays when tracking. For example, if targets move in a sinusoidal or elliptical pattern, cursor–target phase lags are significantly shortened and may be eliminated entirely (Poulton 1952b; Stark et al. 1961; Stepp 2009; Stepp and Turvey 2017; Viviani and Mounoud 1990). In contrast, during tracking of a pseudorandom or sum-of-sines signal (the addition of several sinusoids of different amplitudes and frequencies), delays are observed to be in the region of 180–200 ms (Khoramshahi et al. 2014; Parker et al. 2017; Viviani et al. 1987; Yu et al. 2014)—considerably longer than the plausible minimum feedback times observed in reaching experiments. This phase delay difference between periodic and non-periodic targets likely represents the employment of different control strategies in the PPC, where target and cursor spatial and motion comparisons are thought to occur (Hill and Raab 2005). While the long-phase delay of pseudorandom target tracking appears to indicate a feedback control mechanism, the reduction in phase delay when tracking periodically repeating targets appears to indicate a strategy supporting the anticipation of the target position.

One of the earliest researchers of anticipatory tracking behavior, Poulton (1952b), distinguished between two plausible mechanisms to estimate future target position: speed anticipation and course anticipation. Speed anticipation uses local velocity or acceleration to update position estimates, while course anticipation uses longer term regularities in the target pattern. Course anticipation may require an internal model of target pattern and of plant dynamics. Phase synchronization by phase-locked oscillators may account for course anticipation for sinusoidal targets with a known amplitude (Stepp and Frank 2009; Stepp and Turvey 2017; Voss 2000; Voss et al. 2007). Tracking of more complex pseudorandom targets can also be improved by course anticipation, but only if the target pattern is tracked multiple times. Repeated pseudorandom segments are tracked more accurately than novel segments, even in cases in which participants are not aware the segment has been repeated (Ewolds et al. 2017). Course anticipation may therefore follow implicit learning of the segment rather than an explicit or intentional reproduction of the target pattern. However, as phase lags were not measured in this study, it is not clear whether the performance improvement was due to a reduction in phase lag. Unlike course anticipation, speed anticipation does not require an explicit or implicit internal model of the target pattern. Instead, local velocity information is exploited to bias the outdated estimate of target position toward its future position. Speed anticipation by extrapolation of target velocity has been found to underpin oculomotor and motor behaviors such as ocular smooth pursuit (Khoei et al. 2013; Soechting et al. 2010), manual object interception (Brenner and Smeets 2015; Dessing et al. 2005; Soechting et al. 2009), and visual and manual tracking across brief occlusions (Fine et al. 2014; Mrotek and Soechting 2007; Zago et al. 2010). Perceiving and accounting for target acceleration information during tracking could confer additional control capability when velocity is changing. Interestingly, it does not appear that target acceleration information is used in this way during manual interception (Soechting et al. 2009). This may be due to longer feedback delays for acceleration than for position and velocity (Bennett et al. 2007; Brouwer et al. 2002b).

Due to the dependence of course anticipation on an internal model of the target pattern, it cannot be utilized when tracking complex, non-repeating targets if they have not previously been observed. In contrast, speed anticipation does not require an internal model of the target pattern. As such, speed anticipation could be used irrespective of whether targets have any repeating or periodic characteristics. Despite this potential for the universal application of speed anticipation during tracking, the observation of long-phase delays during pseudorandom target tracking appears to indicate a speed anticipation strategy is not employed during tracking non-periodic targets (Rohde et al. 2014). In the current study, we employed a model-based analysis of tracking behavior to establish whether participants exploit local velocity information during tracking (speed anticipation).

To investigate whether target velocity is exploited to help anticipate target movements during tracking, we fit two models to pseudorandom and sinusoid tracking data. One model controlled only target–cursor positional difference by negative feedback. This model is the canonical perceptual control model (Powers 1978, 2008) which has been extensively used in previous experiments; see Parker et al. (2020) for a review. The second model simulated a speed anticipation strategy by extrapolating target position based on its velocity, and then controlling for the difference between this estimate and the cursor position. To evaluate the capacity for models to compensate sensorimotor delays, models were fit and evaluated at a range of loop delay values. This was the model equivalent to previous studies in which participants’ feedback delays were artificially extended by delaying the effect of the joystick on the on-screen cursor position (Foulkes and Miall 2000; Miall and Jackson 2006; Rohde et al. 2014; Vercher and Gauthier 1992), as loop delay determined the interval of sampling delay in estimates of position and velocity. We expected that comparison of the simulation results across the range of delay values would elucidate the degree to which speed anticipation is likely to have been utilized as a tracking strategy for the two target types. Our hypotheses were as follows:

1. Following previous experimental evidence, we expected sinusoid targets to be tracked with increased accuracy (RMSE) relative to pseudorandom as a result of a reduced phase lag compared with pseudorandom targets. We did not expect a significant difference in amplitude ratio between the target types.
2. We expected that pseudorandom tracking would be simulated most accurately at a loop delays greater than 100 ms (the plausible minimum estimate of sensorimotor feedback time). We were agnostic with respect to whether the models would differ in simulation accuracy.
3. We expected that the position control model would simulate sinusoid tracking most accurately at implausibly low loop delay values. In comparison, the position extrapolation model should simulate sinusoid tracking data most accurately at delays greater than 100 ms, indicating a compensation of sensorimotor delay by target extrapolation.

## Method

### Design

**Fig. 1:**
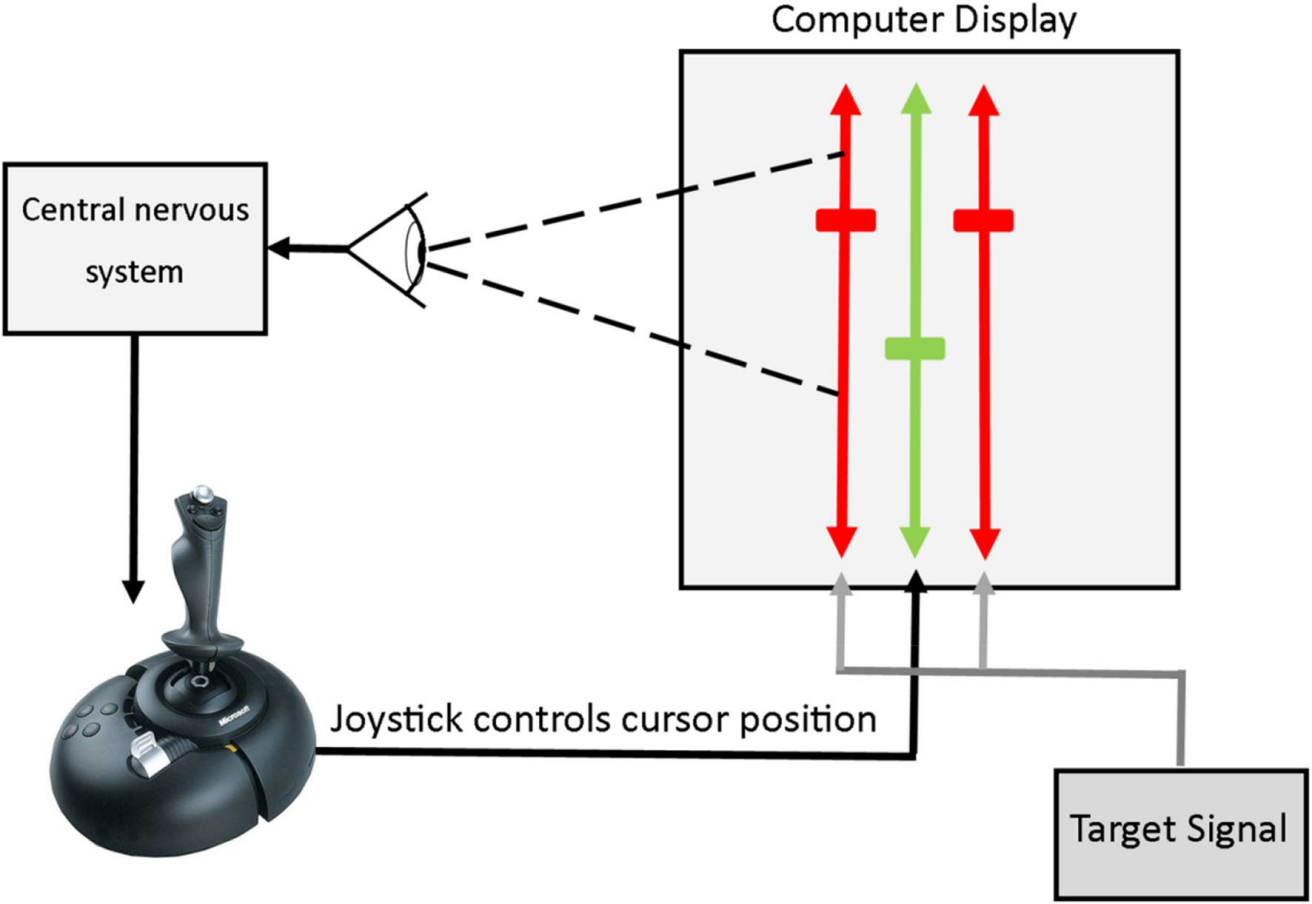
Computerized pursuit manual tracking task set-up, adapted from (Parker et al. 2020)*.* The participant was instructed to keep the target marks (red) and cursor marks (green) aligned during a 1 min trial. The participant controlled the joystick to affect cursor position. The target marks moved according to a target signal. The target signal was either sinusoidal or pseudorandom

In the experiment, participants completed trials of a pursuit tracking task (Fig. 1). In the task, a joystick is used to move a cursor to track a target that moves in the vertical dimension on the screen. The target pattern was either a single sinusoid or continuous pseudorandom signal and the trial duration was 1 min. Participants completed three blocks of 15 pursuit tracking trials. The first block of trials were practice trials, aimed to stabilize participant performance. The second and third blocks were analyzed and are reported in this article. All three blocks were completed in a single experimental session.

Participants were randomly allocated to one of two conditions that specified the volume of training in each target type and the order in which the targets were tracked. In the first condition pseudorandom–sinusoid (PS), participants tracked pseudorandom targets in blocks one and two, and tracked sinusoid targets in block three. In the second condition, sinusoid–pseudorandom (SP), participants tracked sinusoid targets in blocks one and two, and pseudorandom targets in block three. Each block comprised 15 trials. In the two test blocks, trials 1–5 were considered additional practice trials. Trials 6–15 were analyzed (and later used to optimize and validate models). Even-numbered trials (6, 8, 10, 12, and 14) were optimization trials, while odd-numbered trials (7, 9, 11, 13, and 15) were validation trials.

### Participants

Thirty adult volunteers were recruited via the university volunteer database and were reimbursed for their participation either monetarily or with course credits. Individuals were unable to participate if they had been diagnosed with neurological motor impairments or uncorrected visual impairments. No recruited individuals met these criteria and so none were excluded. Ethical approval was granted by the university ethics committee.

Participant data were collected within the same recruitment cycle as within a previous study (Parker et al. 2017). This data collection cycle recruited 80 participants to four experimental conditions. There was no crossover in participant data analyzed between this study and the previous study. The previous study analyzed data from one condition (20 participants) and the current study analyzed data from two other conditions (40 participants). Of these 40 participants, 30 completed the practice trials at the same difficulty level and, therefore, similar target frequencies. These 30 participants were included in the analysis as their models could be directly compared. Due to this split in the experimental data between two separate research articles with different hypotheses, no power analysis was conducted for the current article. However, the sample size for the current experiment is substantially larger than comparable and recently published manual tracking and modeling studies which used between 10 and 22 participants (Gollee et al. 2017; Inoue and Sakaguchi 2014; Stepp and Turvey 2017).

### Apparatus

#### Pursuit tracking task

Pursuit tracking data were collected in TrackAnalyze, a software program developed by Powers (2008), programmed in Pascal. The pursuit tracking task was adapted by the author Abbott for this experiment. In TrackAnalyze, a cursor can be controlled by a joystick or mouse to follow the target. Targets can move in a pseudorandom or single sinusoid pattern and the difficulty can be manipulated by the frequency of the component sinusoids. For each trial, target time series were generated within TrackAnalyze. The algorithm initialized three variables (*D*1, *D*2, and *D*3) to 0. Random numbers were generated between 0 and 1 (Rand), normalized around zero. These pseudorandom numbers were smoothed by dividing each component number by one of five smoothing factors (64, 32, 16, 8, and 4, respectively). These smoothing factors determine the difficulty of the task by altering the rate of change of the target. This process is displayed in Eq. (1):

1 $$D1_{\left( t \right)} = D1_{{\left( {t - 1} \right)}} + \frac{{\left( {{\text{Rand}} {-} 0.5} \right)}}{{{\text{Smooth}}}}.$$

This process was repeated a further twice:

2 $$D2_{\left( t \right)} = D2_{{\left( {t - 1} \right)}} + \frac{{\left( {D1_{\left( t \right)} {-} D2_{{\left( {t - 1} \right)}} } \right)}}{{{\text{Smooth}}}},$$

3 $$D3_{\left( t \right)} = D3_{{\left( {t - 1} \right)}} + \frac{{\left( {D2_{{\left( {t - 1} \right) }} {-} D3_{{\left( {t - 1} \right)}} } \right)}}{{{\text{Smooth}}}}.$$

The *D*3 values were then scaled to have a range of − 500 to 500 and mean 0. The purpose of this was to rescale the numbers to screen size in pixels. Sinusoid targets required no smoothing. Difficulty was manipulated by changing the frequency of the sinusoid. This is computed in the following manner:

4 $$D_{\left( t \right)} = \sin \left( {\frac{t \times 2\pi }{{{\text{Slowing}}}}} \right) ,$$

5 $$D_{\left( t \right)} = D_{\left( t \right)} \times \frac{{{\text{Range}}}}{2}.$$

The slowing factors were 120, 240, 480, 960, and 1920, or 2, 4, 8, 16, and 32 cycles per minute. Equation (5) normalized the sine wave to vary between − 500 and 500 screen pixels. This range of 1000 pixels accounted for 19 cm of on-screen displacement.

The cursor and target positions were sampled every 1/60th of a second (~ 16.67 ms). At the end of each 1-min trial, the sampled cursor and target positions were saved to a tab delimited text file. While the TrackAnalyze program has a simple built-in analysis tool, we analyzed the data for this study in Mathworks Matlab (2019).

#### Joystick

The joystick that the participants controlled was a Microsoft Sidewinder Force Feedback 2 computerized joystick. The force feedback functionality was turned off, such that participants tracked without force cantering. The angle of the joystick determined the position of the cursor on the screen, the full range of movement of the joystick was scaled the maximum displacement of the cursor on the screen.

#### Procedure

Participants first completed the Edinburgh Handedness Inventory (Veale 2014). Participants were first provided with written and oral instructions for the manual tracking task and were given the opportunity to ask questions. Participants completed one practice trial on each of the target patterns. Following which they completed 15 practice tracking runs according to the condition to which they were assigned (Block 1). Participants then completed the first test block using the same type of target that they had tracked in practice (Block 2). A 5-min break followed the end of Block 2. The second test block was then completed (Block 3). This involved 15 tracking trials of the target type that was not tracked in the previous two blocks. A new pseudorandom pattern was generated for each trial for each participant, while the sinusoid differed only in start point.

### Analysis

**Fig. 2:**
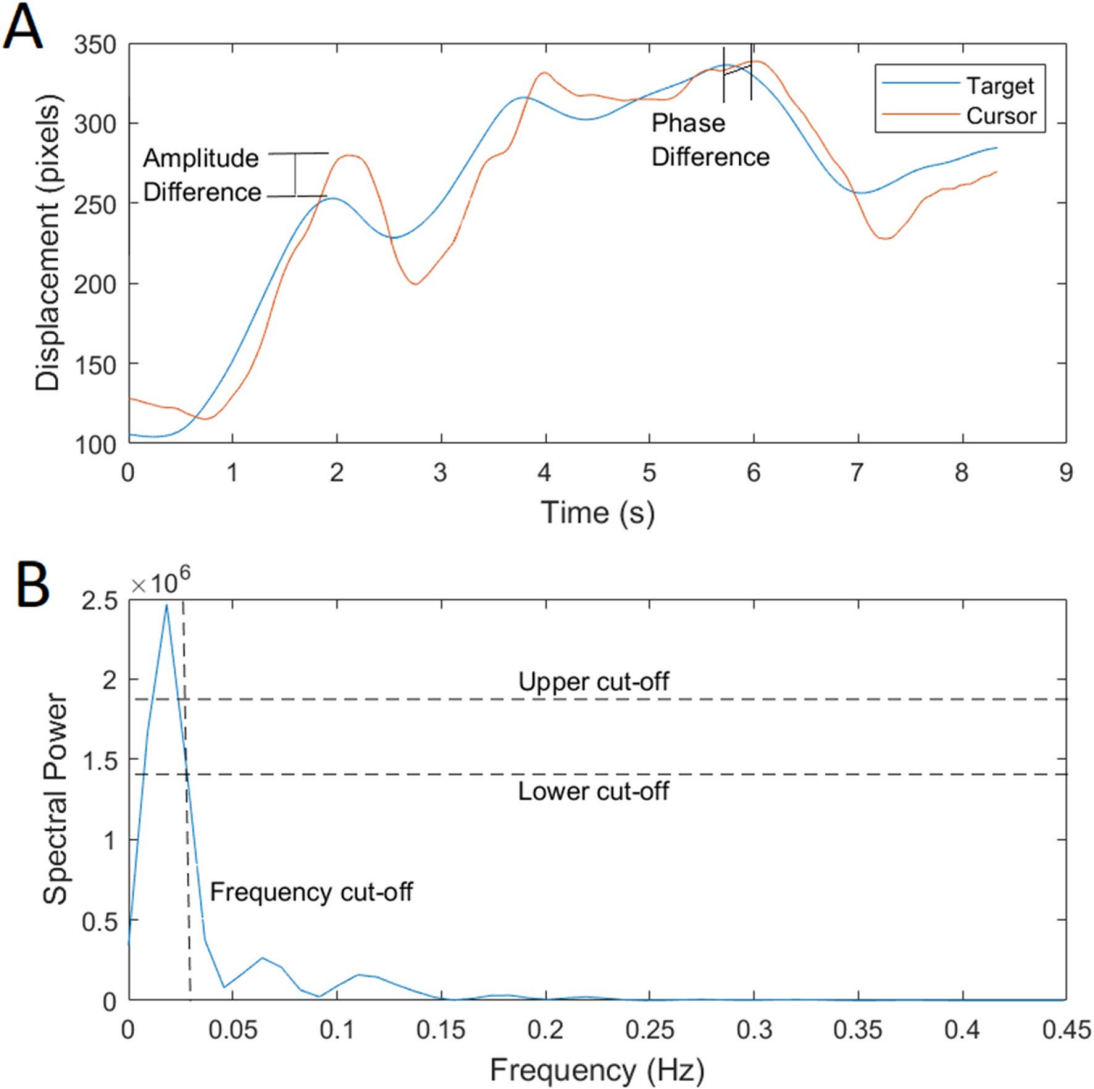
A 9-s segment of a tracking trial showing amplitude and phase difference (**a**) and the spectral power of the target signal plotted over frequency with the cut-offs used in the calculation of amplitude and phase displayed

Time-series and frequency-domain analysis were used to generate accuracy statistics Overall tracking accuracy was calculated by the root-mean-square error (RMSE) in position and velocity. As the response delay was of primary interest to our hypotheses, frequency-domain analysis was conducted to disambiguate errors due to timing and those due to force production. We therefore calculated phase delay: the average delay of the cursor relative to the target across the trial, and amplitude ratio: the difference in displacement between the target and cursor (Fig. 2, panel a).

The frequency analysis was conducted according to the procedure of a previous paper (Cofré Lizama et al. 2013), with several minor adaptations. We designed custom software for this purpose within Matlab. This software used the Welch algorithm with a window length of 0.25 times the length of the signal, and an overlap of 0.9 times the window length. Signals were resampled by fast Fourier transformation resampling to achieve a resolution in the frequency domain of 0.0017 Hz. In practice, this required a scaling factor of ten times the original target signal length, an effective change in the sampling rate from 1/60 to 1/600. As all sinusoid targets had a single frequency, the statistics were calculated at this frequency only. For pseudorandom targets, the signal comprised many frequencies. To analyze amplitude and phase across the frequencies, we followed the procedure of a previous study (Cofré Lizama et al. 2013). The fundamental elements of the procedure are displayed in Fig. 2, panel b. The mean of the three frequencies with the largest power values was calculated. The value 0.75 times this mean determined the lower cut-off. The frequency at which the power fell below the lower cut-off was the cut-off frequency. These constraints defined the band of frequencies over which the phase and amplitude measures were calculated. Phase and amplitudes for each trial were calculated by meaning the statistic across frequencies within this band. Across all pseudorandom trials, the mean frequency from all pseudorandom signals was 0.0248 Hz, range [0.0141–0.0751 Hz]. The frequency of the sinusoid targets was 0.0624 Hz.

The phase statistic reflects the degree of temporal asynchrony between the cursor and target at their maximal values, a positive value would show that the cursor is, in general, advanced of the target in time, while a negative value would represent a phase lag; the cursor lagging the target in time. An amplitude ratio of 1 equals an equal ratio of cursor-to-target displacement. Values above 1 show an increase in cursor amplitude relative to the target. Amplitude ratios are not affected by phase delay.

Following previous findings of reduced phase lag for sinusoid tracking, we hypothesized that participants’ tracking accuracy would be superior for sinusoid targets than for pseudorandom targets, due to reduced phase error for the sinusoid targets. Mixed ANOVAs were conducted to detect differences in tracking accuracy on each of the four criteria: root-mean-square error in position and velocity, amplitude ratio, and phase lag. The repeated-measures factor in each analysis was target type and had two levels: pseudorandom and sinusoid. The independent factor was order and had two levels: pseudorandom–sinusoid (PS) and sinusoid–pseudorandom (SP). Metrics were calculated by the mean value for each participant across 10 trials (five estimation and five validation trials). All data manipulation and analyses were completed in Mathworks MatLab (2019) and JASP (0.11.1.0). Estimation and validation trials were generated by the same processes and parameters [pseudorandom: Eqs. (1–3); sinusoid: Eqs. (4 and 5)].

### Data modeling

Two computational models were developed and evaluated. These were tested on the optimization trial data and evaluated by their fit to the validation trial data.

#### Position control model

**Fig. 3:**
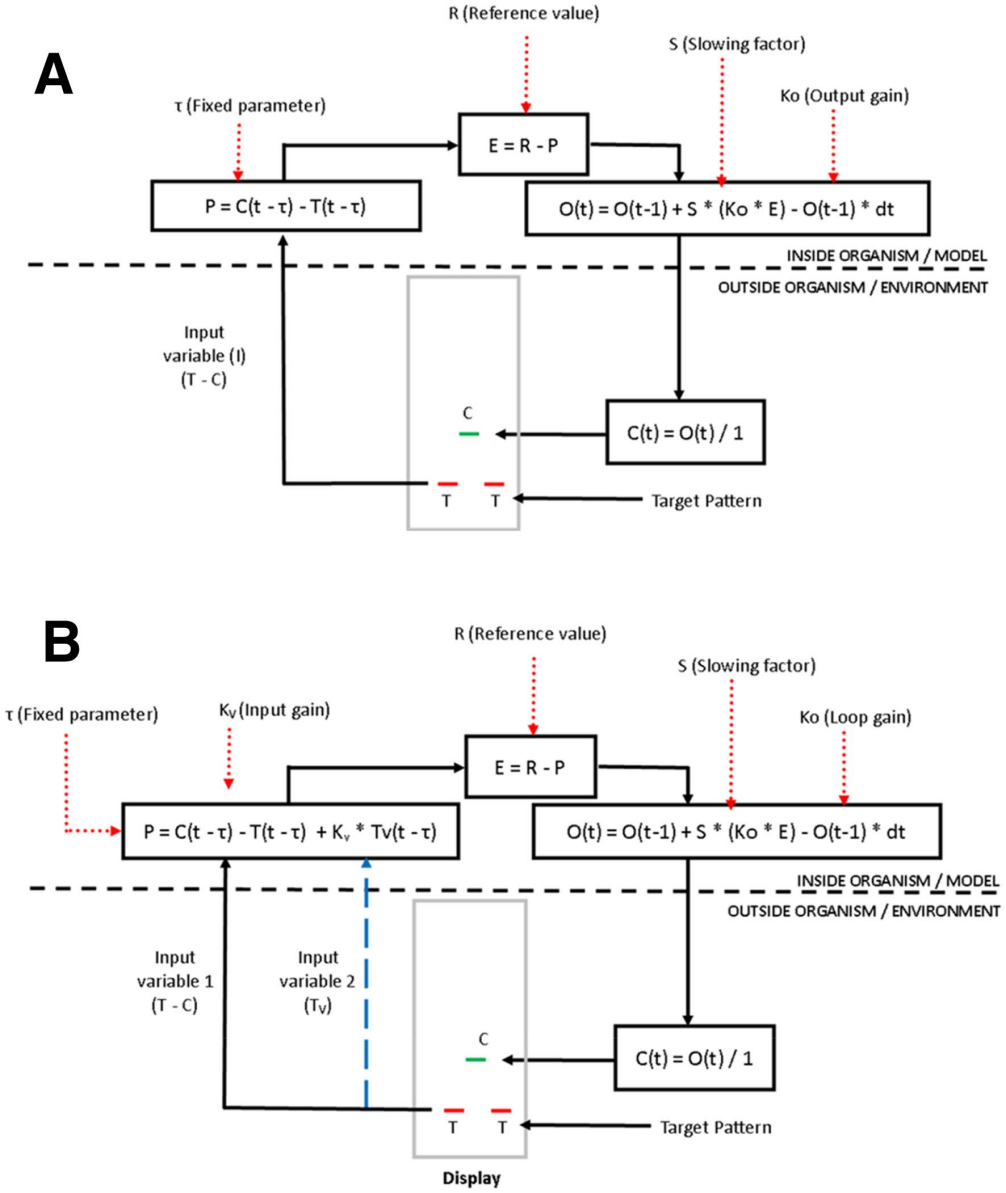
Diagram of the position control model (**a**) and position extrapolation model (**b**)

The first computational model used for comparison in this experiment was the standard tracking model proposed by perceptual control theory (PCT; Powers 1973, 2008). A flow diagram of the model is displayed in Fig. 3, panel a. The model iteratively computed outputs every sample. The current sample in the equations is denoted by (*t*). The inputs to the model are the target (*T*) and cursor (*C*) positions. The perceptual signal (*P*) was the difference between the target and cursor positions, sampled with a loop delay (*τ* samples). Equation (6):

6 $$P = C_{{\left( {t - \tau } \right)}} {-} T_{{\left( {t {-} \tau } \right)}} .$$

The perceptual signal was compared to the reference signal (*R*), which represents the intended difference to be kept between the target and cursor. It might be expected that the value of the reference signal was zero as participants were instructed to maintain zero distance between the cursor and target. The comparison yields the error term (*E*: Eq. 7):

7 $$E = R {-} P.$$

This error signal was fed into the output function where the output (*O*) was computed via Eq. (8):

8 $$O_{\left( t \right)} = O_{{\left( {t - 1} \right)}} + S \times \left[ { K_{{\text{o}}} \times E {-} O_{{\left( {t - 1} \right) }} } \right] \times {\text{ d}}t.$$

In this equation, *O*(*t*) is the current output and *O*(*t* *−* 1) is the output at the previous iteration. The loop gain, *K*_o_, is a proportional gain and accounts for gains on the input, output, and environment functions. *S* is the slowing factor which is the gain for the leaky integrator and determines the proportion of the response that is lost per iteration. The sample rate, d*t* was set to the sample rate: 1.67 ms. The loop delay parameter (*τ*) determines the temporal delay in sampling input and approximates the sensorimotor delay. The loop delay parameter was sequentially manipulated according to our hypotheses, but was fixed during optimizations, while the other parameters (*K*, *S*, *R*) were free parameters. The output of the system determined cursor position (*C*(*t*)) directly as the environment function gain was unity (Eq. 9):

9 $$C \left( t \right) = O_{\left( t \right) } / 1.$$

#### Position extrapolation model

The position control model was adapted, such that the perceptual control variable combined target velocity in target position. By biasing position with velocity, the model would control to an extrapolated target position, and reduce the response delay between the target and cursor (Fig. 3, panel b). Another PCT control model has been previously reported in a conference paper that may function similarly (Taylor 1995). In the position extrapolation model, the inputs to the model are cursor (*C*) and target (*T*) position and target velocity (*Tv*). Cursor velocity is not included in the model as the phase delay is not a result of a difference in target–cursor velocity, but a constant offset in position that must be reduced by a temporary increase in cursor velocity (exceeding the target velocity). Thus, the position extrapolation model controls to a target position advanced of its perceived location.^1^

The perceptual signal (*P*_V_) combines these inputs with a gain (*K*_v_) that determines the weighting between velocity and position inputs; Eq. (10):

10 $$P_{{\text{V}}} { } = C_{{\left( {t - \tau } \right)}} - T_{{\left( {t - \tau } \right)}} + \left[ { K_{{\text{v}}} \times T_{{{\text{v}}\left( {t - \tau } \right)}} } \right].$$

Following this altered perceptual signal, the model loop functions identically to the position control model (Eqs. 7 through 9).

#### Parameter estimation and validation

The loop delay parameter was manipulated sequentially between the values of 1.6 ms and 481.6 ms in 8 ms intervals. The models were optimized at each loop delay value for each trial (60 optimizations per trial). Excepting the loop delay value (*τ*), all other model parameters were free parameters for estimation. The parameters of the model were estimated with the MatLab function *lsqnonlin*, a non-linear least-squares algorithm at each loop delay value. The maximum number of iterations was set to 2000, and the function tolerance to 1 × 10^–8^. The initial conditions and boundaries for parameter optimization were: output gain (*K*), 1 [1, 200]; slowing factor (*S*), 0 [0, 1]; reference value (*R*), 0 [− 500, 500], and extrapolation gain (*K*_V_), 0 [0, 200].

For parameter estimation trials, the parameters of the models were estimated at each delay value on each trial. The set of model parameter values that resulted in the best fit to the cursor movements (lowest RMSE) was selected as the individual model for that specific delay value for each participant. These parameter combinations were used for model validation at each of the delay values.

Validation trial data were simulated and the accuracy of fit to the individual’s movements was assessed at each of the loop delay values. Statistical outliers in model fit were identified by RMSE values greater than three standard deviations above the mean error rate for that participant at that loop delay value. For any outlying data, the next best-fitting model parameters from optimization would be selected and used to simulate the validation data again. This process would continue till model fit statistics were no longer outlying. In practice, outliers occurred infrequently when they did occur, and second best parameter combinations did not produce outliers.

### Model analysis

Our second and third hypotheses aimed to evaluate which of the two models simulated tracking performance more accurately when loop delays were greater than 100 ms. Model simulation accuracy was assessed by three measures, identical to those used to analyze participants’ tracking accuracy (see section entitled tracking analysis). Root-mean-square error measured overall simulation error (accuracy). These errors were disambiguated by frequency analysis. We calculated the amplitude ratio, phase delay, and coherence between the simulated cursor and target, and the simulated cursor and participant cursor. All statistics were calculated for each model following fitting at each delay value. These were 60 values between 1 and 495 ms in 8.33 ms intervals. Results for the fit to validation data are reported in this article, because validation trials provide a more robust test of the models as target signals are not identical to those that the model was trained on (Oberkampf et al. 2004). The model fit criteria are presented with 95% confidence intervals and optima highlighted, indicating the statistical robustness of the comparisons at each delay value.

## Results

### Tracking results

**Fig. 4:**
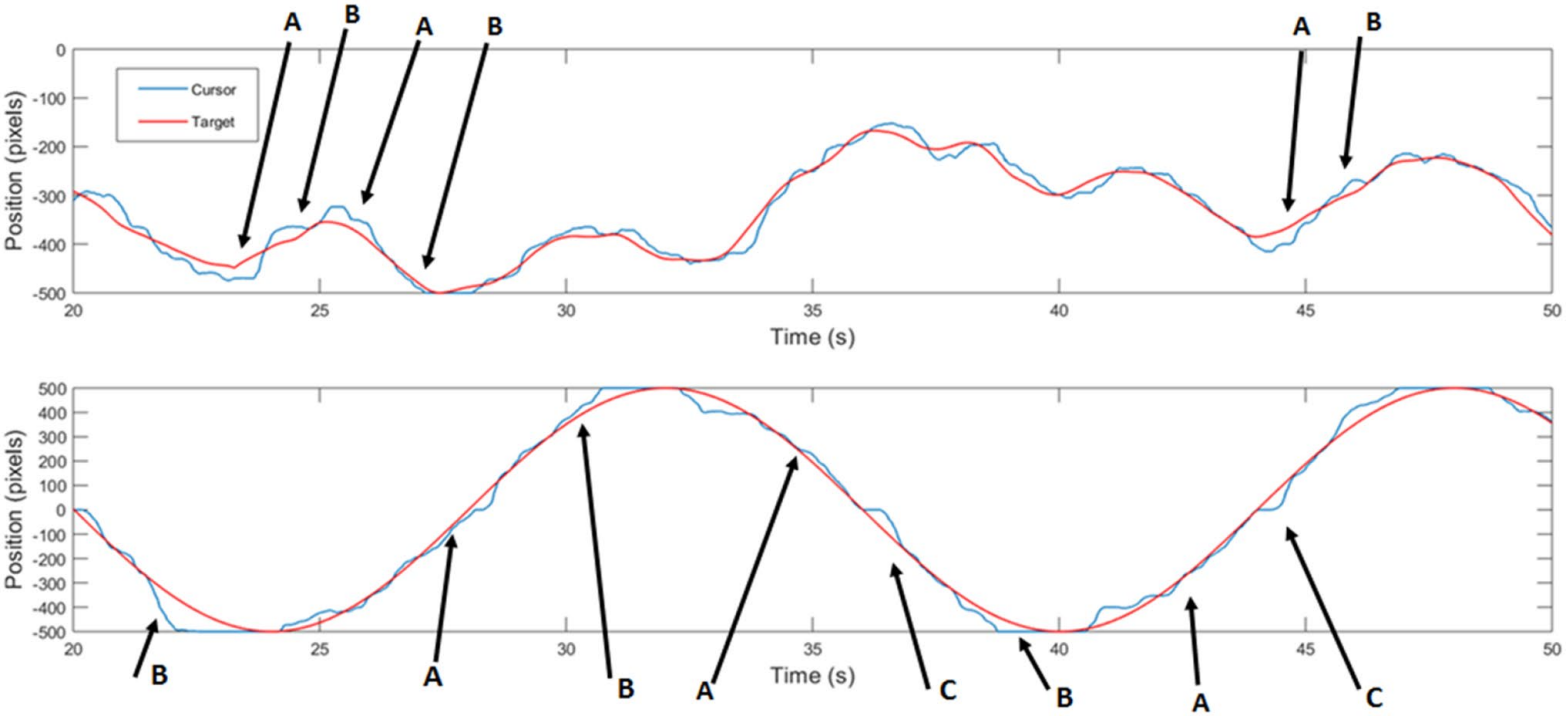
Example segments of tracking trials for a pseudorandom target (top) and sinusoid target (bottom) from the same participant

Two participants were excluded from the analysis as they had missing data, and so 28 participants’ data were analyzed. Typical target and cursor movements during tracking of pseudorandom trial and sinusoid trials are displayed in Fig. 4. Note that, in this figure, each diagram has a different range on the y axis. The displacement of the sinusoid is 1000 pixels. This was not always the case for pseudorandom targets. The longer phase delay for pseudorandom targets is visually apparent. Finally, note the alternating pattern of phase delay during target acceleration (A in diagram) and phase advance during target deceleration (B in diagram), which may be indicative of velocity-based extrapolation. C indicates where joystick stickiness in the center may cause a small displacement when tracking across the centerline.

### Differences in tracking accuracy and phase delays

**Fig. 5:**
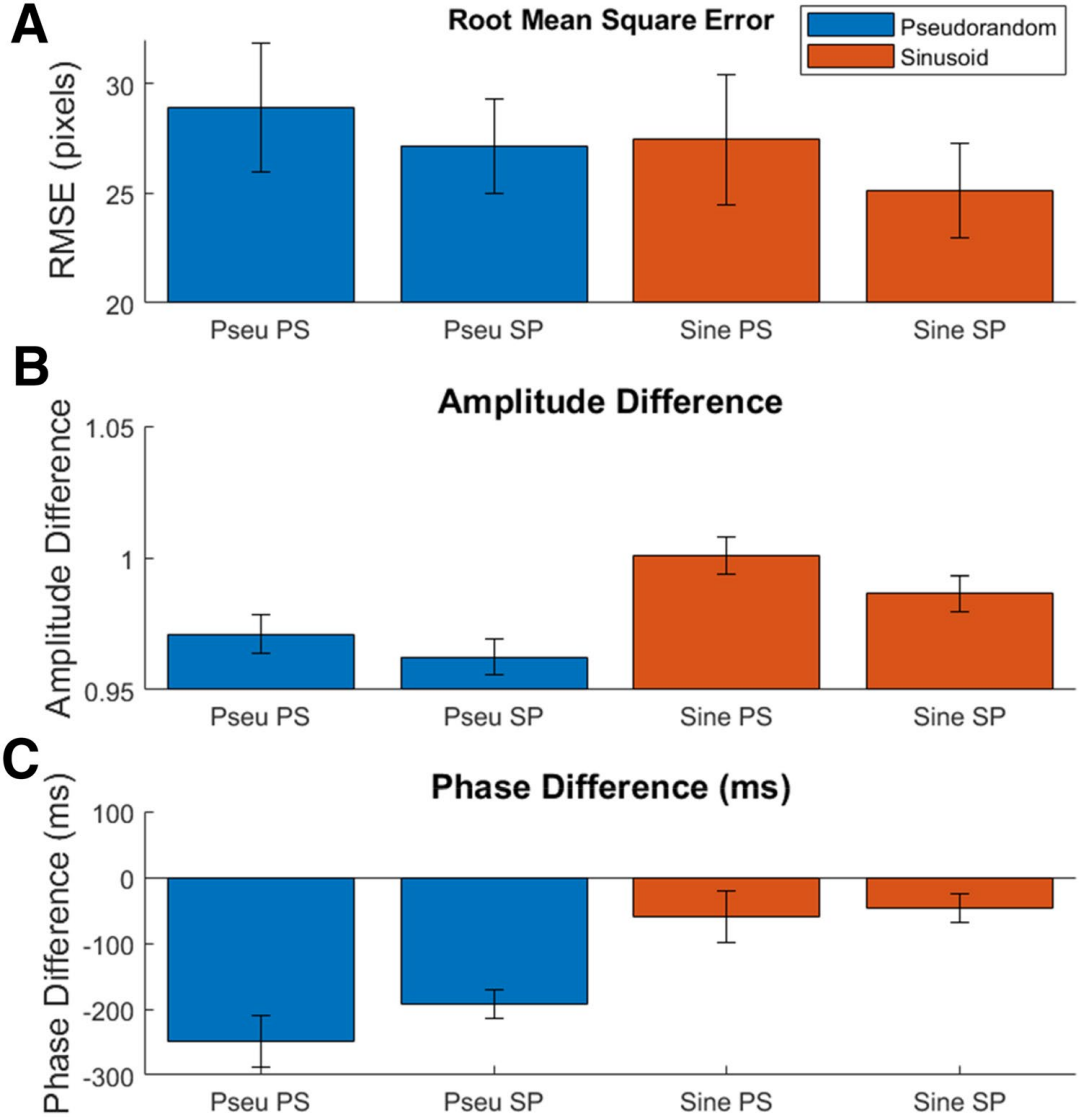
Bar graph of target–cursor tracking statistics (mean and paired standard error) for each target type and order: **a** root-mean-square error in position (in pixels), **b** amplitude ratio, and **c** phase difference, expressed in milliseconds. An amplitude ratio of 1 would indicate that the participant cursor displacement is equal to the displacement of the target. A negative amplitude ratio indicates cursor displacements smaller than the target displacement (undershoot). A phase difference of 0 indicates temporal alignment of the cursor with the target. Negative values indicate phase lags of the cursor relative to the target. Paired standard error is calculated across target type (within each order grouping)

The means and standard errors for the four tracking accuracy criteria are displayed in Fig. 5. The comparison of cursor–target root-mean-square (Fig. 5, panel a) error indicated no overall difference in tracking accuracy between pseudorandom (*M* = 28.03, SD = 6.01) and sinusoid targets (*M* = 26.28, SD = 4.95) or between orders, nor an interaction between them; target type: *F*(1,25) = 0.91, *p* = 0.348, *η*^2^*G* = 0.015; order: *F*(1,25) = 0.91, *p* = 0.349, *η*^2^*G* = 0.020; interaction: *F*(1,25) = 0.03, *p* = 0.872, *η*^2^*G* = 0.000. In contrast, frequency-domain analysis revealed differences in both amplitude and phase.

Comparison of target–cursor amplitudes (Fig. 5, panel b) was significantly reduced for pseudorandom targets relative to sinusoid targets; *F*(1,25) = 28.95, *p* < 0.001, *η*^2^*G* = 0.319. There was a trend toward greater cursor amplitude for participants in the PS group, though this did not reach significance; *F*(1,25) = 3.89, *p* = 0.059, *η*^2^*G* = 0.080. There was no interaction of target type and order; *F*(1,25) = 0.32, *p* = 0.578, *η*^2^*G* = 0.005. As there was no effect of order, amplitudes were pooled across order for the one-sample *t* tests. These revealed that cursor amplitudes were significantly smaller than target amplitudes for both pseudorandom (*M* = 0.96, SD = 0.03) and sinusoid targets (*M* = 0.99, SD = 0.02); pseudorandom: *t*(27) = 7.09, *p* < 0.001, *d* = 1.34; sinusoid: *t*(27) = 2.07, *p* = 0.049, *d* = 0.39.

Comparison of the phase lag (Fig. 5, panel c) between the target and cursor for each target type and order revealed that pseudorandom targets were tracked with a significantly greater delay than sinusoid targets; *F*(1,25) = 56.07, *p* < 0.001, *η*^2^*G* = 0.481. There was no effect of order nor an interaction between target type and order, order: *F*(1,25) = 1.90, *p* = 0.180, *η*^2^*G* = 0.040; interaction: *F*(1,25) = 0.96, *p* = 0.336, *η*^2^*G* = 0.016. As there was no effect of order on phase lag, one-sample t tests pooled means across order. These revealed that participant cursors significantly lagged behind targets for both pseudorandom (*M* = − 220.86 ms, SD = 109.78 ms) and sinusoid targets (*M* = − 53.56 ms, SD = 28.39 ms); pseudorandom: *t*(27) = 9.47, *p* < 0.001, *d* = 1.79; sinusoid: *t*(27) = 7.82, *p* < 0.001, *d* = 1.48.

### Model results

Simulation of validation data generated outlying data for two participants, both for pseudorandom target trials. For one participant, this happened on a single trial for both models and these were replaced non-outlying data produced by simulation with the next best-fitting model parameters from optimization for that participant. For the other participant, all five validation trials for the position control model produced highly abnormal data, and thus, the participant was excluded for both models and both target types.

#### Simulation accuracy for pseudorandom targets

**Fig. 6:**
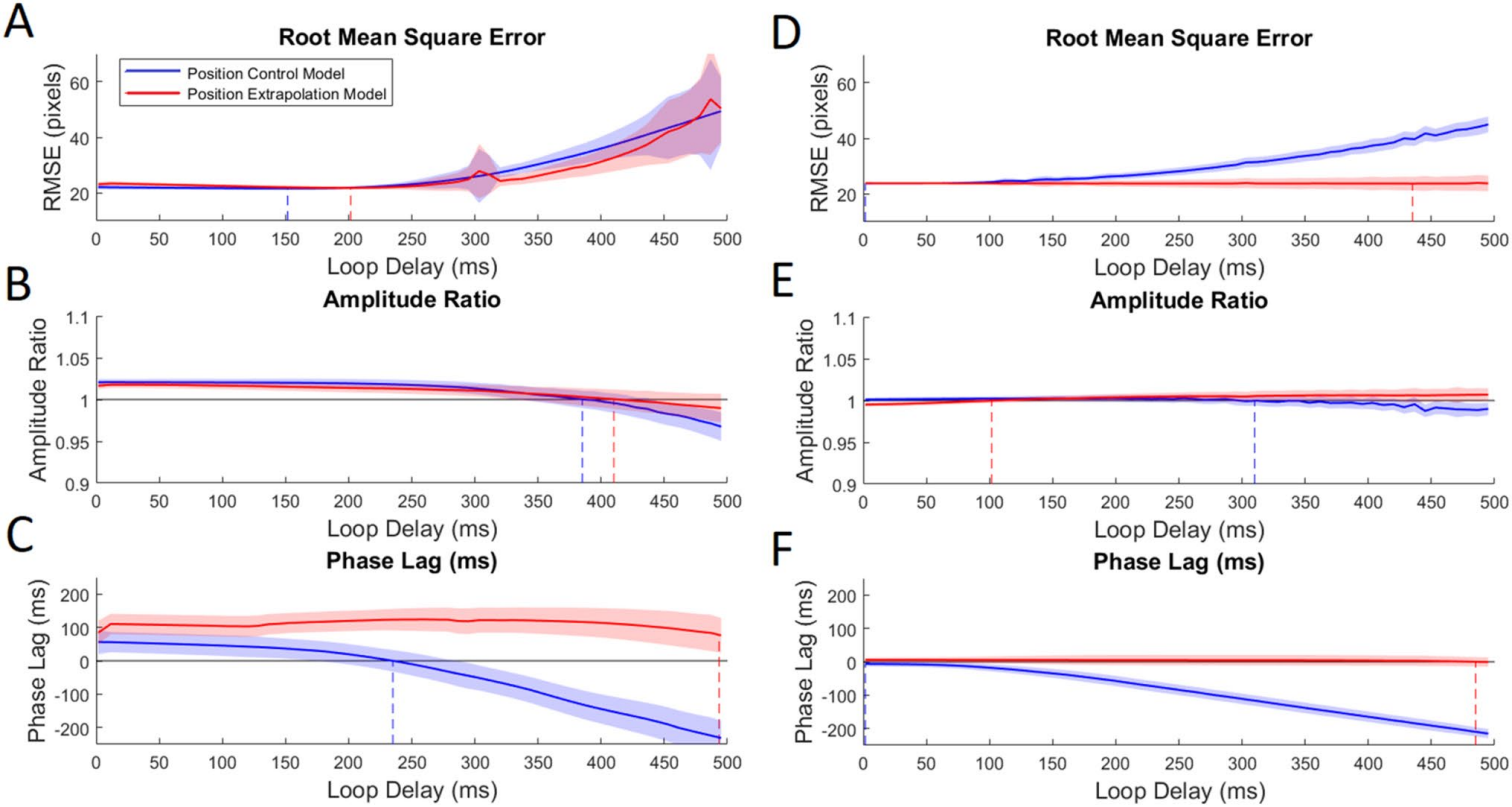
Graphs compare key model simulation accuracy statistics for each model and target type. The blue line indicates the position control model, while the red line is the position extrapolation model. Model accuracy statistics for the pseudorandom target are presented in the left panel and for sinusoids in the right panel. Solid lines display the value of the statistic for each model optimized at each loop delay value; shaded lines indicate 95% confidence interval for the statistic value. Regions of overlap therefore suggest regions of no significant difference between models

On inspection of the RMSE data (Fig. 6, panel a), optima for the position control model and position extrapolation model were found at 152 and 202 ms at which points the error rates were equivalent at 21.62 and 21.82, respectively. However, neither the model showed a clear optimum RMS value as this remained almost constant from 0 ms until around 250 ms for both models, there were no differences between the models in this range. Following 250 ms, mean model accuracy began to diverge as simulation error increased for both models. However, these differences were not significant. Amplitude ratios (Fig. 6, panel b) also remained equivalent for both models and never deviated significantly from one another. Both models began with a value greater than 1, indicating that the model simulated the overshot in participants’ cursors initially, and then, amplitude ratios fell to around unity at 385 ms (position control model) and 410 ms (position extrapolation model). With regard to phase differences (Fig. 6, panel c), the position model produced an initial positive phase advance of around 50 ms; this decreased as a function of loop delay and best simulated the phase delay of the participants at 235 ms. The position extrapolation model also produced a phase advance relative to the participant cursor with a magnitude of approximately 100 ms. This was maintained or slightly increased over the range of loop delays until dropping at long loop delays to a minimum of 76 ms phase advance at 495 ms loop delay.

#### Simulation accuracy for sinusoid targets

We expected that sinusoid target tracking would be simulated more accurately by the position extrapolation model at loop delays longer than 100 ms. Root-mean-square error (Fig. 6, panel d) was initially equivalent and began to diverge significantly after 170 ms loop delay. At longer loop delays, the position control model simulated participant cursor movements with increasingly greater error than the position extrapolation model. The simulation accuracy of the position extrapolation model remained flat across loop delay values. The optima were found at extreme loop delay values. For the position model, the optimal delay was 1.67 ms, whereas for the position extrapolation model, the optimal loop delay value was found at 425 ms; these optima were 23.85 and 23.71, respectively. Amplitude ratios (Fig. 6, panel e) remained around 1 for both models and only significantly deviated from one another prior to 60 ms and later than 360 ms. In both cases, the amplitude ratio for the position extrapolation model was closer to 1. Phase difference (Fig. 6, panel f) differed significantly between models from 85 ms loop delay with the position extrapolation model reproducing participant cursor lags with greater accuracy. At the shortest loop delay values, the position control model simulated cursor movements with around 10 ms of phase delay, whereas the position extrapolation model simulated cursors with around 5 ms phase advance. The phase of the position extrapolation model did not deviate significantly across loop delay values, remaining between 6 ms of phase advance and 1 ms of phase delay. In contrast, the phase lag of the position control model’s simulated cursor relative to the actual cursor movements decreased substantially across the range of loop delay values to a maximum of 215 ms phase lag.

## Discussion

### Differences in tracking accuracy and phase delays

We aimed to investigate whether humans exploit local target velocity when tracking of pseudorandom and sinusoid targets. We hypothesized, based on previous findings, that sinusoid targets would be tracked with superior accuracy to pseudorandom targets and that sinusoids would be tracked with reduced phase lag. We did not clearly observe that sinusoids were tracked with greater overall accuracy (assessed by RMSE). However, analysis of errors in timing revealed that sinusoid targets were tracked with a shorter phase lag than pseudorandom targets, and reduced amplitude error. Thus, while overall model simulation accuracy was not significantly greater for sinusoid targets, the reduction in phase lag and amplitude error when tracking sinusoids indicate participants were tracking with reduced timing delay and undershoot. Critically, the phase lag for sinusoid targets was substantially shorter than the minimum estimates of sensorimotor feedback delays (Brenner and Smeets 2015; Day and Lyon 2000; Foulkes and Miall 2000; Franklin and Wolpert 2008; Saunders and Knill 2005). In comparison, phase delays for pseudorandom targets were well within a range consistent with visual feedback estimates. These findings suggest that when tracking sinusoid targets, sensorimotor delays are compensated, presumably by exploiting target signal properties, but that this may not be the case when tracking pseudorandom targets. Phase lags for both pseudorandom and sinusoid targets corroborated the previous estimates of phase lags (Gollee et al. 2017; Inoue and Sakaguchi 2014; Khoramshahi et al. 2014; Neilson et al. 1993; Parker et al. 2017; Poulton 1952a; Stepp and Turvey 2017; Viviani et al. 1987; Viviani and Mounoud 1990; Yu et al. 2014).

### Simulation accuracy for pseudorandom targets

We had hypothesized that pseudorandom tracking performance would be most accurately simulated at loop delays longer than 100 ms. This was predicted on the basis that pseudorandom targets are tracked with a sufficiently long-phase delay to suggest recruitment of feedback, and previous findings of optimal loop delay parameter values for position control models simulating pseudorandom tracking (Parker et al. 2017). While RMSE optima were greater than 100 ms and so appeared to confirm the hypothesis, RMSE did not significantly reduce or increase over the range of 0–250 ms for either model. This indicates that the parameters of both models could be altered to compensate for increasing loop delays across this range with no decrement to fit. A better model fit for the position extrapolation model could indicate that participants utilized target extrapolation within their control strategy. As no significant difference was found over the range of loop delays, we did not find evidence that participants’ exploit target velocity information in this way during pseudorandom target tracking. This conclusion was supported by the other accuracy measures. Phase-lag optima indicated that the position control model simulated cursor behavior with less timing error than the position extrapolation model until around 400 ms of delay, within a plausible range of sensorimotor delay times. In fact, the position extrapolation model consistently produced a phase lead relative to the cursor, indicating that the model over-compensated participants’ sensorimotor delays. Amplitude ratios did not differ between models across loop delay values.

Considering the results together, it appears that there was little difference in accuracy between models for pseudorandom targets. Any differences in model simulation accuracy were accounted most by differences in timing and not displacement—in which the position control model better emulated participants’ cursor phase lag between 100 and 300 ms, and the difference reversed thereafter. Thus, it appears that exploiting local velocity to extrapolate target position confers no additional advantage for models simulating pseudorandom targets unless sensorimotor delays are very long, supporting previous findings that sensorimotor delays were not compensated during tracking of pseudorandom target signals (Rohde et al. 2014). It appears improbable that participants use such a control strategy when tracking these targets.

### Simulation accuracy for sinusoid targets

We hypothesized that the position control model would suffer decrement to simulation accuracy with increasing loop delays. This was considered likely as participants tend to track sinusoids with a reduced phase lag, indicative of sensorimotor delay mitigation, and the position control model has no capability to mitigate delays. This appeared to be the case as the simulation accuracy decreased as a function of increasing loop delay for the position control model. In contrast, we predicted that the position extrapolation model would fit optimally at loop delays above the plausible minimum sensorimotor delay (100 ms). This was not the case. Instead, the accuracy of the position extrapolation model remained almost constant across the whole range of loop delay values. However, the fit of the position extrapolation model was statistically superior to the position control model for all values above 170 ms.

The simulated cursor of the position control model lagged the participants’ cursor at all loop delay values and this lag increased as a function of input delay, confirming that the position control model did not compensate feedback delay. In comparison, the position extrapolation model maintained a small (< 6 ms) phase advance relative to participants’ cursors across the range of delay values, and began to significantly outperform the position control model in emulating participants’ cursor lags at feedback delays of 85 ms, indicating that the position extrapolation model more effectively mitigated feedback delays even at the shortest biologically feasible estimates of sensorimotor feedback delay (100 ms; Saunders and Knill 2005). Both models simulated the participants’ cursor displacement (amplitude ratio) accurately across a broad range of loop delay values. Taken together, the results indicated that target velocity information may be exploited to benefit tracking accuracy for sinusoids by mitigating sensorimotor delays, and that the improvement in fit is driven by reduced errors in timing rather than displacement. This benefit appears present from implausibly fast feedback delays (< 100 ms in phase-lag measure) and increases in magnitude as a function of feedback delay duration even long delays are mitigated with little decrement to model fit, supporting the interpretation that longer feedback delays can be compensated effectively when tracking sufficiently regularly moving targets.

### General discussion

We evaluated two models, a position control model and a model that controlled to an extrapolated position, biased by local velocity. The results indicated that participants may track regularly moving targets, such as sinusoids, by employing a ‘speed anticipation’ strategy—using the delayed target velocity to extrapolate target position—a strategy that has been identified in other motor and oculomotor tasks (Brenner and Smeets 2015; Brouwer et al. 2002a, b; Dessing et al. 2009; Lisberger et al. 1987; Mrotek and Soechting 2007; Pavel et al. 1992; Soechting et al. 2010). A key conclusion from this study is that a negative-feedback control system, of the kind specified by perceptual control theory, can effectively compensate for sensory delay when tracking a visual object to a similar extent as human participants do when tracking sinusoids. The existence of a significant sensory delay is a key argument put forward that negative-feedback control is insufficient and requires an internal model of the tracked object that can be used to predict and plan actions ahead of their execution (Scott 2008; Wolpert et al. 1998). This claim has been challenged on several fronts (Mansell 2020; Powers 1978; Taylor 1999). The current study illustrates that at least in the case of manual tracking of a visual object in one dimension, a simple negative-feedback system that controls a velocity-biased position input signal can mitigate delays and simulate human ‘anticipatory’ tracking of predictable targets. Future research will clarify the conditions under which this parsimonious strategy is employed and if there exist conditions in which an internal model is necessary.

Interestingly, the position extrapolation strategy does not appear to be used when tracking targets that move in a less predictable pseudorandom pattern, despite the availability of local velocity information in this context. It is yet unclear why speed anticipation is not used in pseudorandom target irrespective of target type, as acceleration and velocity of the target are reliable indicators of the future position of the target while in motion. One possible explanation is that the direction of motion following a change in direction is always predictable for the sinusoid target, whereas for pseudorandom targets, the target may change direction uncertainly following target stoppage, and thus cannot be determined by the participant in advance. This may contribute to participants’ inability to use of anticipatory strategies for pseudorandom targets. Similarly, the likelihood of target directional change is increased as the target approaches the edge of the screen. Sinusoid targets regularly approach these extrema, while pseudorandom targets reach these extrema less often as they changed direction at a randomized set of vertical positions. These predictable elements of the target trajectory could be harnessed locally, prior to a change in direction of the sinusoid target, to anticipate the target change point and direction by using local cues. This conclusion is supported by the findings of another study which found that when a change in target direction was predictable, participants showed an ‘anticipatory’ positive peak in SMA activity that averaged 170 ms before the target changed direction (Hill 2014; Hill and Raab 2005). However, when the change could not be determined from the target trajectory, this peak shifted to follow the change in direction and tracking latencies were increased (Hill 2009). Both local trajectory-relevant cues and local velocity information may be used without explicit representation of the target pattern.

Notably, the benefit of extrapolation was most evident in longer delays for both target types. This may help to explain the ability for participants to maintain tracking accuracy with artificially extended feedback delays, as has been observed in several studies (Foulkes and Miall 2000; Vercher and Gauthier 1992). It may be the case that positional feedback control may be augmented with speed anticipation to maintain accuracy in the presence of longer than human feedback delays like those experienced when operating some machinery.

While the current article advances a control strategy of motion extrapolation, it is also possible that participants use a different control strategy when tracking sinusoid targets. As stated previously, course anticipation may be used to emulate the shape of target pattern in the sinusoid condition. There is evidence that this is the case, particularly when tracking high-frequency targets (Leist et al. 1987; Neilson et al. 1993). With changing acceleration, delayed feedback sampling may become very inaccurate. Thus feedback strategies and speed anticipation may be preferred only at low target speeds. Beyond this point, course anticipation may be used instead if this is more robust to the inaccuracy of sampling due to delays. Alternate strategies could be investigated using the test for the controlled variable (TCV; Mansell and Marken 2015; Marken 2009, 2013, 2014; Runkel 1990). This method applies disturbances to potential control variables. Controlled variables will be protected from these disturbances by compensating action. This could be applied to human control systems in the tracking task. Nevertheless, the existence of a single, generalizable solution to the tracking problem may be unrealistic. It is likely that humans, with their array of sensory inputs, memory, and cognitive abilities, can learn and adapt to perform accurately under different task demands. Modeling of the learning process goes beyond the remit of the current study. In PCT, a learning algorithm known as reorganization develops input functions for controlled variables (in this case position) by adjusting the relative weighting of afferent signals (which in this case would include sensed velocity) through a random-walk process to reduce cumulative error during motor control (Powers 2008).

Many contemporary models posit probabilistic inference to account for noise in inputs and noise in sensorimotor processing (Gollee et al. 2017; Miall et al. 1993; Miall and Jackson 2006). Research on the ‘flash-lag’ effect in manual interception has led to a model based on a probabilistic representation of velocity information to explain position coding (Khoei et al. 2013, 2017). However, other studies have refuted the contribution of probabilistic inference in tracking (Soechting et al. 2009; Zago et al. 2010). The current study adds to the evidence that participants may improve tracking performance using negative feedback control on a perception of extrapolated position without probabilistic internal models. However, as we make no direct comparison between the extrapolation model and probabilistic models, nor test the extrapolation model under conditions of noise, we make no claim regarding the utility of probabilistic inference in manual tracking.

On a methodological note, in both tracking and model results, RMSE did not appear consistently sensitive to errors due to amplitude ratio or phase. For example, sinusoid targets a significant phase difference in the models was present from 85 ms, whereas differences in RMSE were only significant at 170 ms. It should be noted that, for the current investigation, the phase measure was a more suitable and precise measure of the hypothesized behavior, sensorimotor delay compensation, than RMSE. Experimenters should similarly use spectral analysis in addition to RMSE to disambiguate the causes of tracking error.

## Conclusions

We simulated human tracking of repeating and non-repeating patterns with two control models, one using only feedback of target and cursor positions, and the other also utilizing target velocity information to anticipate target motion. Both tracking and simulation results appear to indicate that participants exploit local target velocity when tracking targets that move in repeating patterns. Conversely, we did not find evidence that participants use this strategy when non-repeating, pseudorandom targets—despite the availability of velocity information in both cases.

## Footnotes

1 A spreadsheet demonstration of a simplified position control model and position extrapolation model tracking a constant velocity target signal and a sinusoid target signal can be accessed here: https://docs.google.com/spreadsheets/d/1_Nt5eCapZIqcz7KH1n1wuzIs2jrDymfZF5l0TrZfBLY/edit?usp=sharing.
